# The Changing Phenotype of Inflammatory Bowel Disease

**DOI:** 10.1155/2016/1619053

**Published:** 2016-12-06

**Authors:** Carthage Moran, Donal Sheehan, Fergus Shanahan

**Affiliations:** Department of Medicine and the APC Microbiome Institute, University College Cork, National University of Ireland, Cork, Ireland

## Abstract

It is widely known that there have been improvements in patient care and an increased incidence of Inflammatory Bowel Disease (IBD) worldwide in recent decades. However, less well known are the phenotypic changes that have occurred; these are discussed in this review. Namely, we discuss the emergence of obesity in patients with IBD, elderly onset disease, mortality rates, colorectal cancer risk, the burden of medications and comorbidities, and the improvement in surgical treatment with a decrease in surgical rates in recent decades.

## 1. Introduction

The incidence of Inflammatory Bowel Diseases (IBD) is increasing worldwide [1]. There continues to be remarkable changes in the incidence of IBD amongst different ethnic groups in recent decades as they are exposed to increasingly industrialized environments [2]. Less well known are the phenotypic changes that have occurred in patients with IBD (see Table 1) [3].

## 2. Disease Phenotype

Disease phenotype at diagnosis of IBD has changed in recent decades [3]. A Danish study, investigating consecutive population based cohorts, describes these changes: the proportion of Crohn's disease (CD) amongst total IBD cohort increased and the prevalence of CD and ulcerative colitis (UC) patients who were smokers at diagnosis decreased with time. The median age at diagnosis was stable over five decades for CD patients but increased from 34 years to 38 years in patients with UC [4].

A Dutch population study of patients newly diagnosed in 2006 found that 61% of patients with CD had ileal involvement, 31% had stricturing or penetrating disease [5]. Mean age at diagnosis for CD patients was 36.7 years. In the Olmsted County cohort study (1970–2004), 64% of patients at diagnosis had ileal involvement and 18.6% complicated disease [6]. The phenotype at diagnosis in patients with UC is generally split equally between proctitis, left sided disease, and pancolitis [7, 8]. The proportion of patients presenting with pancolitis increased over the last five decades in Denmark [4].

The phenotype of disease amongst Asian patients with IBD has been described to differ from that of patients from North America and Western Europe [9]. Male predominance [10] increased ileocolonic disease has been described amongst Asian cohorts of patients with CD. However a prospective study failed to show significant difference in disease location between Australian and Asian cohorts [8] (see Table 2 for disease location at diagnosis amongst different geographical cohorts). A follow-up study of this cohort revealed that disease behavior for Asian patients with CD can be as severe as in the West [11].

IBD in patients with Primary Sclerosing Cholangitis (IBD-PSC) represents a distinct phenotype. There is a male predominance, with three-quarters of patients having coexisting UC and PSC [12]. In patients with UC there is an increased risk of pancolitis [12] and subsequent pouchitis (not related to the severity of liver disease) [13] and colorectal cancer [14], in addition to risks of cholangiocarcinoma, liver failure, and gallbladder cancer. In patients with coexisting CD and PSC, colonic disease is present in the vast majority of patients [12]. Prolonged duration of IBD is associated with an increased risk of cholangiocarcinoma in patients with IBD-PSC [15].

## 3. Obesity

Malnutrition has long been recognized as a complication of IBD. Previously attention was focused on patients who were underweight, but obesity is increasingly associated with IBD [16]. Obesity has reached epidemic proportions in western countries, becoming an equal if not greater contributor to burden of disease than smoking in the United States [17]. Regression in life expectancy in the 21st century is predicted if the rate of obesity goes unabated [18].

A Scottish study reported that the prevalence of obese and overweight patients in an IBD population was 18% and 38%, respectively [19]. In the overweight/obese cohort of UC patients there were higher levels of surgery, but the converse was true for the CD cohort. Interestingly in this study there were significantly more obese patients with CD than UC [19]. A third of patients with IBD in a cohort from Texas were obese [20]. Patients with CD enrolling in clinical trials had an increase in weight and disease activity in the last 20 years [21].

Mesenteric fat has long been shown to be an indicator of regional disease activity in CD. “Creeping fat” [22], or fat wrapping, has been used by surgeons to help identify the most diseased regions of bowel. However the role of obesity in development of IBD is unclear. Adipose tissue is not inert; it is well known to be actively involved in both systemic and intestinal inflammatory responses in patients with IBD [23]. A large prospective study found no association between obesity and development of incident IBD [24]. This study had a predominance of middle-aged subjects. IBD tends to present at an earlier age. Conversely, a recent case control study investigated a cohort of patients aged 50 to 70 years, finding obesity was more common in patients with CD than community controls [25]. A subsequent study found that obese women were at elevated risk of developing CD [26].

Early paediatric IBD cohorts have been described as being underweight and malnourished, with lower BMI than background population distribution [27]. However, more recent studies reveal that children with IBD are affected by current population trends towards weight gain; 20–30% and 10% of UC and CD incident cases were overweight or at risk of being overweight as per BMI [28]. These studies also showed that 7–9% and 22–24% of UC and CD incident cases had a low BMI [28].

A large, multicenter cohort study of children with IBD performed in the US, where childhood obesity is epidemic [29], found the overall prevalence of overweight or obesity in this IBD population to be 23%, with 30% and 20% of UC and CD populations overweight or obese [30]. Paediatric patients with CD who are overweight or obese have higher rates of IBD-related surgery, similar to findings in adult populations [31]. Higher use of corticosteroids was found in the overweight/obese UC (35% vs 27%) but not CD group.

The rise of obesity is especially concerning in patients with IBD as it is a known risk factor for colorectal cancer (CRC) [32] and can affect efficacy of medical treatment, including anti-TNF therapies [33, 34]. Obesity is associated with increased blood loss, operative time, and conversion to open surgery in patients with IBD undergoing laparoscopic surgery [35].

## 4. Elderly Onset IBD

The proportion of the world's population that is elderly is increasing [36]. The prevalence of IBD is increasing worldwide [2], thus managing elderly patients with IBD is an increasing clinical encounter. There is a paucity of literature regarding elderly onset IBD in comparison to “early” onset IBD. This is compounded by the fact that in many clinical trials elderly patients are excluded from study enrollment.

Roughly 10% of patients present with first presentation of IBD aged greater than 65 years [37]. A recent population based cohort study in northern France described the natural history of elderly onset (>60 years) IBD. 5% and 12.5% of incident cases of CD and UC, respectively, were classified as elderly onset over study timeframe (1988–2006). The clinical history at diagnosis and course of disease was milder in elderly onset IBD than younger onset disease [38]. Half of the patients in this large cohort did not undergo surgery nor were exposed to medications other than 5-ASA [38]. Patients with elderly onset IBD had lower IBD-specific healthcare utilization than patients with earlier onset IBD [37]. In contrast, a survey of hospital discharges suggested geriatric IBD patients accounted for a quarter of all IBD-related hospitalizations [39], with higher mortality than younger patients. This study included all patients with IBD older than 65, not just patients with elderly onset IBD [39]. Elderly onset IBD is not associated with an increased risk of intestinal cancer; however there is an increased risk of developing lymphoproliferative (not associated with thiopurine exposure) and myeloproliferative disorders [40].

Elderly patients with acute severe UC (ASUC) had worse outcomes compared to younger patients in audits of IBD care in the United Kingdom. Patients aged greater than sixty years with ASUC had a roughly 4% mortality, compared to 0.1% if younger than sixty [41]. Patients aged greater than eighty with ASUC had mortality rates of 10% [41]. A retrospective Japanese study reported poor outcomes in elderly patients with UC undergoing emergency surgery, with a mortality rate of 27% at 30 days postoperatively for emergency surgery compared to 1% for elective surgery [42].

Colonic CD is more common than small bowel disease or ileocolonic disease in elderly onset disease [43, 44]. There is also a greater tendency for inflammatory, uncomplicated behavior [43] with a relatively low proportion of patients progressing to complicated disease (9%) [38, 45]. Elderly onset UC is characterized by left sided or extensive colitis at presentation, with disease extension rare [38].

In geriatric patients with IBD, longer disease duration is associated with Vitamin D, Vitamin B12, and iron deficiency [46]. Older patients have been shown to have an expedited time to referral, investigation, and diagnosis than younger patients [47]. Other diseases that can mimic symptoms of IBD include diverticulosis, NSAID colitis, microscopic colitis, and ischaemic colitis. Extensive biopsy sampling and vigilance are necessary in order to avoid an erroneous diagnosis purely based on histological mimicry of changes seen in segmental colitis associated with diverticular disease (SCAD), when diagnosing IBD in the presence of diverticulosis coli [48].

## 5. Comorbidities

As the phenotype of IBD changes, so do comorbidities. Studies suggest that fatty liver disease is more common than PSC in patients with IBD [49]. The prevalence of PSC in IBD cohorts is estimated to be less than 5% [49, 50], although a recent study revealed that 8% of patients with long-term IBD screened with magnetic cholangiography had probable PSC (these patients had subclinical PSC, that is, no symptoms and normal liver function tests) [51]. Nonalcoholic fatty liver disease in patients with IBD is common, with estimated prevalence of 8 to 23% [49, 52]. Patients with IBD develop nonalcoholic fatty liver disease (NAFLD) with less metabolic risk factors compared to patients without IBD [52]. The rate of NAFLD likely depends on environmental and genetic risk factors of study population, as a recent prospective Swedish study detected lower rates of NAFLD than American studies [53]. Disease specific risk factors for NAFLD in patients with IBD include small bowel surgery, use of steroids, and disease duration and activity [52, 54]. As the prevalence of NAFLD and subsequently nonalcoholic steatohepatitis induced cirrhosis, increases in patients with IBD, liver failure, and transplantation will complicate management of patients with IBD. Concomitant chronic liver disease doubles the inpatient mortality rate in patients with IBD [55].

It is well known that patients with IBD are at increased risk of developing venous thromboembolism (VTE) [56, 57]. Patients with IBD are at increased risk of developing VTE upon hospital discharge compared with other patients [58]. The inflammatory cascade that increases risk of VTE likely also places patients with IBD at increased risk of heart failure [59] and atrial fibrillation [60] during periods of disease activity. Flares of disease are also associated with increased risk of myocardial infarction, stroke [61], and cardiovascular death [62]. A recent meta-analysis concluded that patients with IBD are at increased risk of ischaemic heart disease, but not of cardiovascular mortality [57]. Further studies are needed to investigate the role of medications and lifestyle factors in patients with IBD to clarify the role their role in the development of cardiovascular disease.

Anxiety and depressive symptoms commonly affect patients with IBD [63] and are associated with body image dissatisfaction [64] and nonadherence to medication [65]. Evidence suggests that treating mood disorders can improve IBD disease activity [66, 67]. Concomitant treatment of mood disorders with antidepressants (multiple classes including tricyclic antidepressants and selective and nonselective serotonin reuptake inhibitors) reduced IBD relapse and steroid use in a retrospective case control study [67]. Tricyclic antidepressants can also improve ongoing GI symptoms in patients with adequate IBD therapy, as defined by their physician [68]. Symptom response was similar in a cohort of patients with IBD compared to group of patients with IBS [68].

## 6. Medication Burden

Patients with IBD are often prescribed multiple medications. Patients with IBD have greater use of antidepressants, sedatives, and analgesics (including narcotic analgesics) than matched controls [78]. Older patients with IBD are at increased risk of polypharmacy and potential adverse medication interactions [79]. An American study found that half of patients with CD met criteria for polypharmacy [80] and that this finding correlated with decreased quality of life and increased disease activity.

Narcotic use was identified in 13% of this cohort, and narcotic use was associated with increased use of other medications, including neuropsychiatric medications [81]. A Canadian population study showed that 5% of patients with IBD will become heavy opiate users within 10 years of IBD diagnosis [82]. An American study found that 70% of patients hospitalized with IBD as primary indication were given narcotics [83]. Factors associated with narcotic use include history of surgery, smoking, and outpatient narcotic use [83]. Healthcare models affect rates of narcotic use amongst patients with IBD, as demonstrated by the variance of narcotic use in different countries and an Australian study, which demonstrated that the introduction of nurse specialists reduced narcotic use amongst patients with IBD [84]. Our anecdotal evidence would be that narcotic use has decreased in recent decades. This is likely secondary to improved medical care and recognition of psychological issues.

## 7. Mortality

There is an increased mortality rate in patients with CD [85, 86] compared to the general population, with conflicting evidence as to relative mortality rates in patients with UC [86, 87]. A population based Canadian study found that there was an increased mortality rate in both CD and UC in the first year after diagnosis compared to the general population, but this only persisted beyond a year in CD and in those who underwent gastrointestinal surgery [88]. A meta-analysis of population based and inception cohorts demonstrated an elevated overall mortality for patients with both UC and CD, with standardized mortality ratios of 1.19 (95% confidence interval, 1.06–1.35) and 1.38 (95% confidence interval, 1.23–1.55) [86]. This meta-analysis revealed that summary mortality rate for CD remained constant over time but improved for UC [86].

A large Danish population study found that mortality in patients with UC decreased over three decades, secondary to decreased mortality from colorectal cancer, suicide, and gastrointestinal disorders [89]. Unfortunately patients with CD were still found to have 50% greater mortality compared to general population, and this did not change over study period of 1982 to 2010 [89]. A diagnosis of PSC was identified as a predictor of premature mortality in an Irish cohort of patients with IBD [90].

## 8. Reduced Risk of Colorectal Cancer?

The risk of CRC in patients with IBD is less than previously reported (meta-analysis of population based studies described a pooled standardized incidence ratio of 1.7 [73]) and is not increased in all patients [91]. The incidence of colorectal cancer (CRC) in patients with UC has decreased in the last few decades [92]. A nationwide Danish cohort found that patients diagnosed with UC in the 1980s were at increased risk of CRC compared to background population; however that excessive risk of CRC has declined and no longer exceeds that of the general population [74]. Extensive colonic disease, concomitant PSC, young age at diagnosis, and longer duration of disease are at increased risk of developing CRC in patients with IBD [73, 74].

Why the risk of CRC is declining in patients is not fully understood. There is conflicting evidence on the role of 5-ASA medications, thiopurines, and biologic medications as chemopreventive agents [93]. Endoscopic surveillance of patients with IBD is recommended, and colonoscopy in preceding three years is associated with reduced incidence of CRC [94]. These finding are in keeping with a German study that found colonoscopy within preceding 10 years was associated with a 77% reduced risk of CRC in the general population [95].

The decrease in gastrointestinal malignancies observed in recent decades has been accompanied by an increase in nongastrointestinal malignancies [96]. There is an increased risk of developing haematological malignancies [91]. Ongoing thiopurine exposure of greater than one year is associated with increased risk of lymphoma in patients with IBD. This elevated risk is not persistent after thiopurines are discontinued [97].

Patients with IBD on immunosuppressive therapy appear to be at increased risk of high grade cervical dysplasia or cancer [98] and melanomatous and nonmelanomatous skin cancer [99, 100].

## 9. Reduced Surgical Rates

The risk of IBD-related surgery has decreased in recent decades [101]; see Table 1 [3]. Advances in medications [71, 72] and specialization of management of patients with IBD [70], in addition to improved surgical techniques, have led to reduced rates of surgery. Further studies on the impact of biological agents on surgical rates are needed.

Surgical intervention remains integral to managing patients with complex IBD and can improve patients' quality of life [102] and should not always be seen as a poor outcome, especially in those with localized disease [103].

## 10. Conclusion

Further studies are needed to investigate the relationship between ongoing chronic inflammation and systemic medications and the development of obesity and cardiovascular disease. Gastroenterologists need to be aware of emerging comorbidities in patients with IBD. Caution is needed in extrapolating results from existing randomized control trials, as most patients would be ineligible to participate in recent trials, whether due to their comorbidities, medication use, or disease phenotype [104].

The increased prevalence of obesity poses a challenge in the management of patients with IBD, not least due to the potential of further increased risk of CRC. Positive lifestyle habits such as exercise and smoking cessation, which may have disease modifying behavior [105], should be encouraged regularly. As the proportion of elderly patients with IBD increases, the potential for polypharmacy and adverse medication interactions also increases. Thus close liaison between gastroenterologists and patient's general physicians needs to remain an integral in the care of patients with IBD.

## Figures and Tables

**Table 1:** The changing phenotype of patients with IBD [3].

Feature	Comment	Reference
Increased BMI	(i) Prevalence of obese and overweight patients in a Scottish IBD population was 18% and 38%, respectively.	[19]
	(ii) 17% of patients with CD in an Irish cohort were obese compared to 12% of controls.	[16]
	(iii) A third of patients with IBD attending metropolitan health services in Texas were obese.	[20]
	(iv) Increased weight of patients with CD enrolling in clinical trials (1991–2008).	[21]
	(v) 23% of paediatric patients with IBD in United States found to be overweight or obese.	[30]

Decreased rate of surgery	(i) Cumulative probability of first major surgery at 9 years decreased from 50% (1979–86) to 23% (2003–11) in patients with CD and 14% to 9% in patients with UC.	[69]
	(ii) Decreased risk of surgery in patients diagnosed with CD after 1996, associated with increased specialist care.	[70]
	(iii) Reduced surgical rates in patients with CD associated with increased and earlier immunomodulator use.	[71, 72]

Reduced proportion of patients with short bowel syndrome	(i) Largely due to improvements and specialization of surgical care. (ii) Partially due to improvements in medical treatments.	Almost a universal observation

Increasing prevalence of elderly onset IBD	(i) Increased proportion of colonic disease and inflammatory behavior, in elderly patients with CD.	[38, 46]
	(ii) Progression of disease behavior less than in younger patients. Milder disease course than younger cohorts.	[38]

Reduced risk of colorectal cancer (CRC) in recent decades	(i) Meta-analysis of population studies found that CRC elevated in patients with IBD, but not as high as previously reported.	[73]
	(ii) Nationwide study in Denmark found that risk of CRC in patients with UC no longer exceeds that of general population.	[74]

**Table 2:** Disease location at diagnosis as per Montreal Classification.

	Hungary [75]	Sweden [76, 77]	Netherlands [5]	Australia [8]	Asia^*∗*^ [8]
*Crohn's disease*					
Ileal	20.2%	28%	21.4%	31%	31%
Colonic	35.6%	49%	36.3%	24%	24%
Ileocolonic	44.2%	23%	38.8%	45%	45%

*Ulcerative colitis*					
Proctitis	26.8%	32%	29%	32%	37%
Left sided colitis	50.9%	31%	52.8%	27%	32%
Pancolitis	22.3%	31%	18.4%	41%	21%

^*∗*^Mainland China, Hong Kong, Indonesia, Macau, Singapore, Sri Lanka, and Thailand.
